# ChatGPT in veterinary medicine: a practical guidance of generative artificial intelligence in clinics, education, and research

**DOI:** 10.3389/fvets.2024.1395934

**Published:** 2024-06-07

**Authors:** Candice P. Chu

**Affiliations:** Department of Veterinary Pathobiology, College of Veterinary Medicine & Biomedical Sciences, Texas A&M University, College Station, TX, United States

**Keywords:** artificial intelligence, AI, generative AI, GenAI, large language model, prompt engineering, machine learning, GPT-4

## Abstract

ChatGPT, the most accessible generative artificial intelligence (AI) tool, offers considerable potential for veterinary medicine, yet a dedicated review of its specific applications is lacking. This review concisely synthesizes the latest research and practical applications of ChatGPT within the clinical, educational, and research domains of veterinary medicine. It intends to provide specific guidance and actionable examples of how generative AI can be directly utilized by veterinary professionals without a programming background. For practitioners, ChatGPT can extract patient data, generate progress notes, and potentially assist in diagnosing complex cases. Veterinary educators can create custom GPTs for student support, while students can utilize ChatGPT for exam preparation. ChatGPT can aid in academic writing tasks in research, but veterinary publishers have set specific requirements for authors to follow. Despite its transformative potential, careful use is essential to avoid pitfalls like hallucination. This review addresses ethical considerations, provides learning resources, and offers tangible examples to guide responsible implementation. A table of key takeaways was provided to summarize this review. By highlighting potential benefits and limitations, this review equips veterinarians, educators, and researchers to harness the power of ChatGPT effectively.

## Introduction

Artificial intelligence (AI) is a trending topic in veterinary medicine. A recent survey on AI in veterinary medicine by Digital and the American Animal Hospital Association, involving 3,968 veterinarians, veterinary technicians/assistants, and students, showed 83.8% of respondents were familiar with AI and its applications in veterinary medicine, with 69.5% using AI tools daily or weekly (1). Yet, 36.9% remain skeptical, citing concerns about the systems’ reliability and accuracy (70.3%), data security and privacy (53.9%), and the lack of training (42.9%) (1).

The current application of AI in veterinary medicine cover a wide range of topics, such as dental radiograph (2), colic detection (3), and mitosis detection in digital pathology (4). Machine learning (ML), a subset of AI, enables systems to learn from data without being explicitly programmed (5). Generative AI (genAI), in turn, is a field within ML specializing in creating new content. As a subset of genAI, large language models (LLMs) are known for their human-like text generation capabilities. Notable LLMs include ChatGPT (OpenAI) (6), which is utilized by Microsoft Copilot for Microsoft 365 (7), Llama 3 (Meta) (8), Gemini (Google) (9), and Claude 3 (Anthropic) (10). ChatGPT, initially powered by GPT-3.5, was made publicly accessible by OpenAI on November 30, 2022 (11). In less than a year, ChatGPT has attracted approximately a hundred million weekly users (12), making it the most popular LLM for newcomers to this technology. Based on PubMed search results, academic articles mentioned ‘ChatGPT’ in the title or abstract grew from 4 in 2022 to 2,062 in 2023, indicating a growing interest in ChatGPT in the medical field (13). Therefore, this review will focus on ChatGPT as the main example of generative AI and discuss its application in veterinary clinics, education, and research.

GPT, or Generative Pre-trained Transformer, excels in *generating* new text, images, and other content formats rather than solely analyzing existing data. It is *pre-trained* by exposure to vast datasets of text and code, enabling it to recognize patterns and generate human-like responses. It employs the *transformer* neural network architecture that is particularly adept at processing language, which enables coherent and contextually relevant outputs (14). The free version of ChatGPT provides the capability of answering questions, providing explanations, generating creative content, offering advice, conducting research, engaging in conversation, supporting technical tasks, aiding with education, and creating summaries. On February 1, 2023, OpenAI released ChatGPT Plus, a subscription-based model later powered by GPT-4, which has capabilities in text, image, and voice analysis and generation (15). OpenAI introduced GPT-4 Turbo with Vision on April 9, 2024 (16). This updated model is accessible to developers through the application programming interface (API). Its ability of taking in images and answer questions has sparked interest in radiology (17, 18), pathology (19), and cancer detection (20, 21). On May 13, 2024, OpenAI released GPT-4o to the public. The ‘o’ in its name emphasizes the new model’s omnipotent in reading, listing, writing, and speaking abilities (22). Despite ChatGPT’s widespread use, a comprehensive review of its applications in veterinary medicine is lacking.

The breadth of ChatGPT in medicine covers a wide range of areas, ranging from answering patient and professional inquiries, promoting patient engagement (23), diagnosing complex clinical cases (24), and creating educational material (25). Searching ‘ChatGPT AND veterinary’ in PubMed yielded 14 results until May 2024. After examining the title and abstract of all articles, 5 articles were deemed relevant to the subject and were included in the review (26–30). In addition, online search using the same combination of keywords identified commercial software that integrated ChatGPT to enhance virtual assistance, diagnostic accuracy, communication with pet owners, and optimization of workflows (31–37). While examples of ChatGPT applications are prevalent on social media and in various publications (38–40), the best way to understand its impact is through direct engagement. This article aims to discuss the applications of ChatGPT in veterinary medicine, provide practical implementations, and examine its limitations and ethical considerations. The following content will use’ ChatGPT’ as a general term. When the information of specific versions of ChatGPT is available, terms such as GPT-3.5 or GPT-4 will be used. Highlights of each section are listed in Table 1 for a quick summary of the review.

**Table 1:** Key takeaways of the review.

**Introduction** Of 3,968 veterinary professionals who participated in a survey, 83.8% of respondents were familiar with AI and its applications in veterinary medicine, with 69.5% using AI tools daily or weekly. Machine learning (ML) is a subset of artificial intelligence (AI) that enables systems to learn from data without being explicitly programmed. Generative AI, in turn, is a field within ML specializing in creating new content. Large language models (LLMs) have human-like text generation capabilities. Examples include ChatGPT (OpenAI), Llama 3 (Meta), Gemini (Google), Gemma (Google), and Claude 3 (Anthropic). GPT stands for Generative Pre-trained Transformer, indicating its characteristics of content generation, pre-trained by text and codes, and the use of transformer neural network. Important milestones of ChatGPT’s public release: November 30, 2022 – ChatGPT (GPT-3.5) February 1, 2023 - ChatGPT Plus (GPT-3.5) March 1, 2023 – ChatGPT (upgrade to GPT-3.5 Turbo) March 14, 2023 – ChatGPT Plus (upgrade to GPT-4) May 13, 2024 – GPT-4o
**ChatGPT 101: prompts and prompt engineering** Prompts act as conversation starters, consisting of instructions or queries that elicit responses from the AI. Prompt engineering is the practice of refining inputs to produce optimal outputs. Common strategies include providing relevant context, detailing the data structure, and specifying desired outcomes. Cognitive strategy prompts can direct ChatGPT’s reasoning more effectively. See Supplementary material.
**Using ChatGPT in clinical care** In human medicine, ChatGPT can make triage decisions, mine text from clinical history, create SOAP notes, diagnose complex cases, and interpret image inputs such as blood work and ECG. A prior publication in veterinary medicine demonstrated ChatGPT’s ability in text-mining. Examples of applying ChatGPT in writing SOAP notes and interpreting ECG and blood work images are available in Supplementary material.
**Using ChatGPT in veterinary education** ChatGPT has the potential to assist medical exam takers, while the performance in standardized exams may vary among different LLMs. GPTs are customized ChatGPT that can serve as an AI tutor for clients and veterinary students. CatGPT: https://chatgpt.com/g/g-NDDXC050T-catgpt VetClinPathGPT: https://chatgpt.com/g/g-rfB5cBZ6X-vetclinpathgpt
**Using ChatGPT in academic writing** Most journal publishers agree that ChatGPT cannot be listed as a co-author. Several veterinary journals request authors to declare the use of ChatGPT in methods, acknowledgment, or designated sections in the manuscript. See Supplementary material. Reviewers could mistakenly classify human writings as AI-generated content, while ML tools built based on specific language features could achieve 99% accuracy in identifying AI-authored texts. The official ‘ChatGPT detectors’ are currently underdeveloped by OpenAI.
**ChatGPT’s limitations and ethical issues** Most veterinary professionals are familiar with AI and its application in veterinary medicine, while some remain skeptical about its reliability and accuracy, data security and privacy, and a lack of training. *Hallucination and inaccuracy* Hallucination, or artificial hallucination, refers to the generation of implausible but confident responses by ChatGPT, which poses a significant issue. See Supplementary material. Inaccuracy is not an uncommon finding when using ChatGPT. These unexpected errors can potentially harm patients. *Intellectual property, cybersecurity, and privacy* ChatGPT is trained using undisclosed but purportedly accessible online data, and user-generated content is consistently gathered by OpenAI. When analyzing clinical data, uploading de-identified datasets is recommended. Alternatively, considering local installations of open-source, free-for-research-use LLMs, like Llama 3 or Gemma, for enhanced security. *U.S. FDA regulation* Most FDA-approved AI and ML-enabled human medical devices are in the field of radiology, followed by cardiovascular and neurology. FDA has not set premarket requirements for AI tools in veterinary medicine. The AI- and ML-enabled veterinary products include dictation and notetaking apps, management and communication software, and radiology services, which may or may not have scientific validation. The continual learning and updating of LLM pose a special regulatory challenge for FDA.
**Practical learning resources** Resources for learning about ChatGPT and generative AI are abundant, including OpenAI’s documentation, online courses from Vanderbilt University via Coursera, Harvard University’s tutorial for generative AI, and the University of Michigan’s guides on using generative AI for scientific research. Links are provided in Supplementary material. Readers are encouraged to ask ChatGPT for learning resources: https://chat.openai.com

## ChatGPT 101: prompts and prompt engineering

Understanding prompts is crucial before engaging with ChatGPT or other generative AI tools. Prompts act as conversation starters, consisting of instructions or queries that elicit responses from the AI. Effective prompts for ChatGPT integrate relevant details and context, enabling the model to deliver precise responses (28). Prompt engineering is the practice of refining inputs to produce optimal outputs. For instance, researchers instructing ChatGPT to identify body condition scores from clinical records begin prompts by detailing the data structure and desired outcomes: “*Each row of the dataset is a different veterinary consultation. In the column ‘Narrative’ there is clinical text. Your task is to extract Body Condition Score (BCS) of the animal at the moment of the consultation if recorded. BCS can be presented on a 9-point scale, example BCS 6/9, or on a 5-point scale, example BCS 3.5/5. Your output should be presented in a short-text version ONLY, following the rules below: … (omitted)* (28)”. Writing effective prompts involves providing contextual details in a clear and specific way and willingness to refine them as needed.

**Figure 1:**
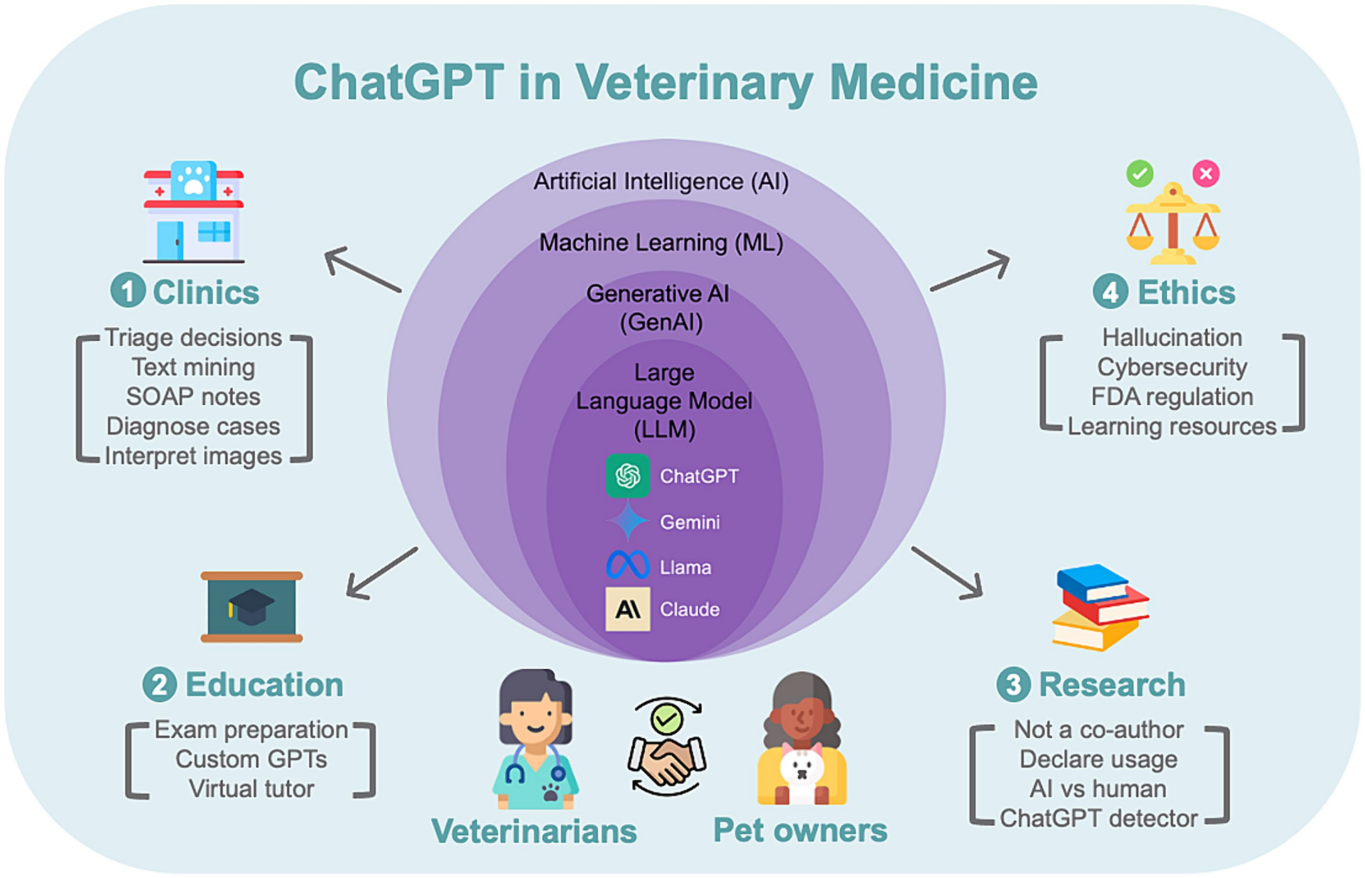
Visual abstract of the review.

Moreover, incorporating ‘cognitive strategy prompts’ can direct ChatGPT’s reasoning more effectively (refer to Supplementary material for more details). For a comprehensive understanding of prompt engineering, readers are encouraged to refer to specialized literature and open-access online courses dedicated to this subject (41–44). Proper prompt engineering is pivotal for shaping conversations and obtaining the intended results, as illustrated by various examples in this review (Figure 1).

## Using ChatGPT in clinical care

ChatGPT has the potential to provide immediate assistance upon the client’s arrival at the clinic. In human medicine, the pre-trained GPT-4 model is adept at processing chief complaints, vital signs, and medical histories entered by emergency medicine physicians, subsequently making triage decisions that align closely with established standards (45). Given that healthcare professionals in the United States spend approximately 35% of their time documenting patient information (46) and that note redundancy is on the rise (47), ChatGPT ‘s ability to distill crucial information from extensive clinical histories and generate clinical documents are particularly valuable (48). In veterinary medicine, a study utilizing GPT-3.5 Turbo for text mining demonstrated the AI’s capability to pinpoint all overweight body condition score (BCS) instances within a dataset with high precision (28). However, some limitations were noted, such as the misclassification of lameness scoring as BCS, an issue that the researchers believe could be addressed through refined prompt engineering (28).

For daily clinical documentation in veterinary settings, veterinarians can input signalment, clinical history, and physical examination findings into ChatGPT to generate Subjective-Objective-Assessment-Plan (SOAP) notes (46). An illustrative veterinary case presented in Supplementary material involved the generation of a SOAP note for a canine albuterol toxicosis incident (49), where ChatGPT efficiently identified the diagnostic tests executed in the case report, demonstrating that ChatGPT can be used as a promising tool to streamline the workflow for veterinarians.

Moreover, recent research has investigated ChatGPT’s proficiency in human clinical challenges. One study found that GPT-4 could accurately diagnose 57% of complex medical cases, a success rate that outperformed 72% of human readers of medical journals in answering multiple-choice questions (24). Additionally, GPT-4’s top diagnosis concurred with the final diagnosis in 39% of cases and included the final diagnosis within the top differential diagnoses in 64% of cases (50). In veterinary medicine, a notable case is a man on social media platform X (previously known as Twitter), who reported that ChatGPT saved his dog’s life by identifying immune-mediated hemolytic anemia—a diagnosis his veterinarian had missed (51). Veterinarians should recognize that pet owners may consult ChatGPT or similar AI chatbots for advice due to their accessibility (26). While the proliferation of veterinary information online can enhance general knowledge among clients, it also risks spreading misinformation (52). Customizing ChatGPT could address these challenges (refer to ‘Using ChatGPT in Veterinary Education’ below).

In a human medicine study, GPT-4 can interpret ECGs and outperformed other LLM tools in correctly interpreting 63% of ECG images (53). A similar study has yet to be found in veterinary medicine. A veterinary example is provided in the Supplementary material, showing that GPT-4 did not identify an atypical atrial flutter with intermittent Ashman phenomenon in a 9-year-old Pug despite the addition of asterisks in the ECG to indicate the wide and tall aberrant QRS complexes (35). This example emphasizes that while ChatGPT is a powerful tool, it cannot replace specialized AI algorithms approved by the Food and Drug Administration (FDA) for ECG interpretation (54, 55). Nevertheless, advances in veterinary-specific AI tools, such as a deep learning model for canine ECG classification, are on the horizon, with the potential to be available soon (56). With the updated image upload function, the capability of GPT-4 and GPT-4o extends to the interpretation of blood work images. The Supplementary material illustrates a veterinary example of GPT-4 and GPT-4o analyzing Case of the Month on eClinPath (57) and providing the correct top differential despite its limited ability to interpret the white blood cell dot plot.

## Using ChatGPT in veterinary education

Recent studies leveraging Large Language Models (LLMs) in medical examinations underscore their utility in educational support. In human medical education, GPT-3’s performance, evaluated using 350 questions from the United States Medical Licensing Exam (USMLE) Steps 1, 2CK, and 3, was commendable, achieving scores near or at the passing threshold across all three levels without specialized training (58). This evaluation involved modifying the exam questions into various formats—open-ended or multiple-choice with or without a forced justification—to gage ChatGPT’s foundational medical knowledge. The AI-generated responses often included key insights, suggesting that ChatGPT’s output could benefit medical students preparing for USMLE (58).

Another investigation in human medical education benchmarked the efficacy of GPT-4, Claude 2, and various open-source LLMs using multiple-choice questions from the Nephrology Self-Assessment Program. Success rates varied widely, with open-source LLMs scoring between 17.1–30.6%, Claude 2 at 54.4%, and GPT-4 leading with 73.7% (59). A comparative analysis of GPT-3.5 and GPT-4 indicates the newer version substantially improved in the neonatal-perinatal medicine board examination (60). In the veterinary education context, researchers at the University of Georgia used GPT-3.5 and GPT-4 to answer faculty-generated 495 multiple-choice and true/false questions from 15 courses in the third-year veterinary curriculum (27). The result concurred with the previous study that GPT-4 (77% correct rate) performed substantially better than GPT-3.5 (55% correct rate); however, their performance is significantly lower than that of veterinary students (86%). These studies highlight the variances in LLM knowledge bases, which could affect the quality of medical and veterinary education.

Beyond exam preparation, the ChatGPT Plus subscribers can create customized ChatGPT, referred to as GPTs (41) that are freely accessible to other users (61). Veterinarians, for instance, can harness these tools to develop AI tutors to educate clients and boost veterinary students’ learning. For client education, the Cornell Feline Health Center recently launched ‘CatGPT,’ a customized ChatGPT that draws information from its website and peer-reviewed scientific publications to answer owner’s inquiries (62). An example of a custom GPT is a specialized veterinary clinical pathology virtual tutor named VetClinPathGPT (63). This custom GPT draws from legally available open-access textbooks with Creative Commons licenses (64–66) and the eClinPath website (57), ensuring the information provided is sourced from credible references. Students are encouraged to pose any question pertinent to veterinary clinical pathology and can even request specific references or links to web pages. More information about this GPT is detailed in the Supplementary material.

## Using ChatGPT in academic writing

The incorporation of AI in academic writing, particularly in the field of medical research, is a topic marked by considerably more controversy than the previous sections discussed. Ever since the development of GPT-3 in 2020, its text-generating ability has ignited debate within academia (67). Leveraging editing services enhances clarity and minimizes grammatical errors in scientific manuscripts, which can improve their acceptance rate (68). While acknowledgments often thank editorial assistance, the use of spelling-checking software is rarely disclosed. Nowadays, AI-powered writing assistants have integrated advanced LLM capabilities to provide nuanced suggestions for tone and context (45), thus merging the line between original and AI-generated content. Generative AI, like ChatGPT, extends its utility by proposing titles, structuring papers, crafting abstracts, and summarizing research, raising questions about the AI’s role in authorship as per the International Committee of Medical Journal Editors’ guidelines (69) (Supplementary material). Notably, traditional scientific journals are cautious with AI, yet NEJM AI stands out for its advocacy for LLM use (70). However, these journals still refrain from recognizing ChatGPT as a co-author due to accountability concerns over accuracy and ethical integrity (70–72). The academic community remains wary of ChatGPT’s potential to overshadow faculty contributions (73).

Several veterinary journals have updated their guidelines in response to the emergence of generative AI. Among the top 20 veterinary medicine journals as per Google Scholar (74), 14 instruct on generative AI usage (Supplementary material). They unanimously advise against listing AI as a co-author, mandating disclosure of AI involvement in Methods, Acknowledgments, or other designated sections. These recommendations typically do not apply to basic grammar and editing tools (Supplementary material). AI could enhance writing efficiency and potentially alleviate disparities in productivity, posing a nuanced proposition that suggests broader acceptance of AI in academia might benefit less skillful writers and foster a more inclusive scholarly community (40).

The detectability of AI-generated content and the associated risks of erroneous academic judgments have become significant concerns. A misjudgment has led an ecologist at Cornell University to face publication rejection after being falsely accused by a reviewer who deemed her work as “obviously ChatGPT” (75). However, a study revealed that reviewers could only identify 68% of ChatGPT-produced scientific abstracts, and they also mistakenly tagged 14% of original works as AI-generated (76). In a veterinary study, veterinary neurologists only had a 31–54% success rate in distinguishing AI-crafted abstracts from authentic works (30).

To counteract this, a ‘ChatGPT detector’ has been suggested. An ML tool utilizes distinguishing features like paragraph complexity, sentence length variability, punctuation marks, and popular wordings, achieving over 99% effectiveness in identifying AI-authored texts (77). A subsequent refined model can further distinguish human writings from GPT-3.5 and GPT-4 writings in chemistry journals with 99% accuracy (78). While these tools are not publicly accessible, OpenAI is developing a classifier to flag AI-generated text (79), emphasizing the importance of academic integrity and responsible AI use.

## ChatGPT’s limitations and ethical issues

### Hallucination and inaccuracy

Hallucination, or artificial hallucination, refers to the generation of implausible but confident responses by ChatGPT, which poses a significant issue (80). ChatGPT is known to create fabricated references with incoherent Pubmed ID (81), a problem somewhat mitigated in GPT-4 (18% error rate) compared to GPT-3.5 (55% error rate) (82). The Supplementary material illustrated an example where GPT-4 could have provided more accurate references, including PMIDs, underscoring its limitations for literature searches.

In the medical field, accuracy is paramount, and ChatGPT’s inaccuracy can have serious consequences for patients. A study evaluating GPT-3.5’s performance in medical decision-making across 17 specialties found that the model largely generated accurate information but could be surprisingly wrong in multiple instances (83). Another study highlighted that while GPT-3.5 (Dec 15 version) can effectively simplify radiology reports for patients, it could produce obviously incorrect interpretations, potentially harming patients (84). With the deployment of GPT-4 and GPT-4o, the updated database should bring expected improvement; however, these inaccuracies underscore the necessity of using ChatGPT cautiously and in conjunction with professional medical advice.

### Intellectual property, cybersecurity, and privacy

As an LLM, ChatGPT is trained using undisclosed but purportedly accessible online data and ongoing refinement through user interactions during conversations (85). It raises concerns about copyright infringement and privacy violations, as evidenced by ongoing lawsuits against OpenAI for allegedly using private or public information without their permission (86–88). Based on information from the OpenAI website, user-generated content is consistently gathered and used to enhance the service and for research purposes (89). This statement implies that any identifiable patient information could be at risk. Therefore, robust cybersecurity measures are necessary to protect patient privacy and ensure compliance with legal standards in medical settings (90). When analyzing clinical data using AI chatbot, uploading de-identified datasets is suggested. Alternatively, considering local installations of open-source, free-for-research-use LLMs, like Llama 3 or Gemma (Google), for enhanced security is recommended (91–94).

### US FDA regulation

While the FDA has approved 882 AI and ML-enabled human medical devices, primarily in radiology (76.1%), followed by cardiology (10.2%) and neurology (3.6%) (95), veterinary medicine lacks specific premarket requirements for AI tools. The AI- and ML-enabled veterinary products currently span from dictation and notetaking apps (34, 35), management and communication software (36, 37), radiology service (31–33), and personalized chemotherapy (96), to name a few. These products may or may not have scientific validation (97–104) and may be utilized by veterinarians despite the clients’ lack of consent or complete understanding. In veterinary medicine, the absence of regulatory oversight, especially in diagnostic imaging, calls for ethical and legal considerations to ensure patient safety in the United States and Canada (105, 106). LLM tools like ChatGPT pose specific regulatory challenges, such as patient data privacy, medical malpractice liability, and informed consent (107). Continuous monitoring and validation are the key, as these models are continuously learning and updating after launch. As of today, FDA has not authorized any medical devices that use genAI or LLM.

## Practical learning resources

Resources for learning about ChatGPT and generative AI are abundant, including AI companies’ documentation (108–110), online courses from Vanderbilt University and IBM on Coursera (41, 111), Harvard University’s tutorial for generative AI (112), and the University of Michigan’s guides on using generative AI for scientific research (113). These resources are invaluable for veterinarians seeking to navigate the evolving landscape of AI in their practice. Last but not least, readers are advised to engage ChatGPT with well-structured prompts, such as: ‘I’m a veterinarian with no background in programming. I’m interested in learning how to use generative AI tools like ChatGPT. Can you recommend some resources for beginners?’ (see Supplementary material).

## The ongoing dialog

In the 2023 Responsible AI for Social and Ethical Healthcare (RAISE) Conference held by the Department of Biomedical Informatics at Harvard Medical School, several principles on the judicious application of AI in human healthcare were highlighted (114). These principles could be effectively adapted to veterinary medicine. Integrating AI into veterinary practices should amplify the benefits to animal welfare, enhance clinical outcomes, broaden access to veterinary services, and enrich the patient and client experience. AI should support rather than replace veterinarians, preserving the essential human touch in animal care.

Transparent and ethical utilization of patient data is paramount, advocating for opt-out mechanisms in data collection processes while safeguarding client confidentiality. AI tools in the veterinary field ought to be envisioned as adjuncts to clinical expertise, with a potential for their role to develop progressively, subject to stringent oversight. The growing need for direct consumer access to AI in veterinary medicine promises advancements but necessitates meticulous regulation to assure pet owners about data provenance and the application of AI.

This review discussed the transformative potential of ChatGPT across clinical, educational, and research domains within veterinary medicine. Continuous dialog, awareness of limitations, and regulatory oversight are crucial to ensure generative AI augments clinical care, educational standards, and academic ethics rather than compromising them. The examples provided in the Supplementary material encourage innovative integration of AI tools into veterinary practice. By embracing responsible adoption, veterinary professionals can harness the full potential of ChatGPT to make the next paradigm shift in veterinary medicine.

## Author contributions

CC: Conceptualization, Project administration, Resources, Visualization, Writing – original draft, Writing – review & editing, Investigation.
