# Supplementary material for: ChatGPT in veterinary medicine: a practical guidance of generative artificial intelligence in clinics, education, and research
Source: Front Vet Sci. 2024 Jun 7;11:1395934. doi: 10.3389/fvets.2024.1395934 (PMC11192069; doi:10.3389/fvets.2024.1395934)
Supplement: Supplementary file 1 [file Table_1.docx]

Supplementary Material

ChatGPT in Veterinary Medicine: A Practical Guidance of Generative Artificial Intelligence in Clinics, Education, and Research

Candice P. Chu^1*^

**^*^ Correspondence:**Candice P. Chu
cchu@cvm.tamu.edu

^1^ Department of Veterinary Pathobiology, College of Veterinary Medicine & Biomedical Sciences, Texas A&M University, College Station, TX, USA

All input prompts provided to a generative AI technology and outputs received from a generative AI technology of this article are available in this supplementary material. When available, the results from different versions of ChatGPT (GPT-3.5, GPT-4, GPT-4o) are provided for comparison. Results are presented in the order they appear in the article with the section subtitle provided.

**Prompts and Prompt Engineering**

| **Common cognitive strategy prompts** | |
| --- | --- |
| - Take a deep breath and think about your answers step-by-step. - Do not respond if you are unsure of the answer. - Ask yes/no questions when possible to limit responses. - Reference sources when necessary. - Ask follow-up questions if you do not understand a task. - Please do an amazing job as my career is on the line. | |
| **Feline diabetes management – comparing GPT-4 and GPT-4o** | |
| **Example without cognitive strategy prompts** | **Example with cognitive strategy prompts** |
| **Prompt**:  How is diabetes managed in cats?  **GPT-4**:  Managing diabetes in cats involves a combination of medical treatment, dietary changes, and lifestyle adjustments to help control blood glucose levels and minimize symptoms. The primary goals are to eliminate the symptoms of diabetes, such as excessive thirst and urination, and to prevent complications. Here are the key components of managing feline diabetes:   1. **Insulin Therapy**: Most diabetic cats require insulin injections to help their bodies use glucose effectively. The type and dosage of insulin are determined by the cat's individual needs and are carefully monitored and adjusted based on blood glucose levels. 2. **Monitoring**: Regular monitoring of blood glucose levels is crucial in managing feline diabetes. This can involve both home monitoring and visits to the veterinarian. Home monitoring can include checking blood glucose levels with a glucometer and observing the cat's general behavior and health. 3. **Dietary Management**: A high-protein, low-carbohydrate diet is often recommended for diabetic cats. This type of diet can help to regulate blood glucose levels. Your veterinarian may recommend specific therapeutic diets formulated for diabetic cats. 4. **Weight Management**: Maintaining an optimal body weight is important in managing diabetes. Overweight cats are more resistant to insulin, so weight loss can improve the body's use of insulin. A balanced diet and regular exercise are key components of weight management. 5. **Regular Exercise**: Regular physical activity can help to improve insulin sensitivity and overall health. Encouraging play and providing opportunities for exercise can be beneficial for diabetic cats. 6. **Routine Veterinary Check-ups**: Regular check-ups with a veterinarian are essential to monitor the cat's diabetes and overall health. These visits may include blood tests, urine tests, and assessments of the cat's response to treatment. 7. **Management of Concurrent Illnesses**: It's important to manage any other health conditions the cat may have, as these can impact diabetes management. Conditions such as dental disease, urinary tract infections, or kidney disease can affect blood glucose levels and the overall health of the cat. 8. **Education and Support**: Caring for a diabetic cat requires a commitment from the cat owner. Veterinary teams often provide education and support to help owners understand the disease, treatment options, and how to manage their cat's condition at home.   It's important to work closely with a veterinarian to develop and adjust a management plan that's tailored to the individual needs of the diabetic cat. With proper management, many diabetic cats can lead happy, active lives. | **Prompt**:  Could you provide a detailed, step-by-step guide on managing diabetes in cats, including dietary recommendations, insulin therapy, and monitoring? I am a veterinarian. It's crucial to get this right for the well-being of the feline patient, so please reference up-to-date veterinary guidelines.  **GPT-4**:  Managing diabetes in cats requires a comprehensive approach that includes dietary management, insulin therapy, and regular monitoring of blood glucose levels. Here's a detailed, step-by-step guide based on current veterinary practices and guidelines:  **1. Diagnosis and Initial Evaluation**   - Confirm the diagnosis of diabetes mellitus through clinical signs and laboratory tests, including blood glucose concentration and urinalysis for glucose and ketones. - Evaluate the cat for concurrent diseases, such as urinary tract infections, chronic kidney disease, or hyperthyroidism, which can complicate diabetes management. - Determine the cat's body condition score and weight to tailor dietary and insulin therapy.   **2. Dietary Management**   - Diet plays a crucial role in managing feline diabetes. The goals are to achieve and maintain ideal body weight and to stabilize blood glucose levels. - High-protein, low-carbohydrate diets are recommended for diabetic cats, as they help improve insulin sensitivity and control blood glucose levels. - Gradual weight loss is important for overweight cats. Aim for 1-2% body weight loss per week to avoid hepatic lipidosis. - Consistent feeding times and amounts are essential, especially if the cat is on insulin. Some veterinarians recommend feeding cats at the time of insulin administration or shortly before.   **3. Insulin Therapy**   - Insulin therapy is often necessary for diabetic cats. The choice of insulin type and dose is based on the individual cat's needs and response. - Long-acting insulin formulations (e.g., glargine, detemir) are commonly used in cats and are preferred for initiating therapy in newly diagnosed cases. - Start with a conservative insulin dose to avoid hypoglycemia. Dosing adjustments should be made based on blood glucose monitoring and clinical response. - Monitor for signs of insulin overdose, such as weakness, lethargy, tremors, or seizures, which require immediate veterinary attention.   **4. Monitoring**   - Home blood glucose monitoring is highly recommended for diabetic cats. It provides more consistent and stress-free readings than in-clinic measurements. - Teach owners how to perform home blood glucose testing using a glucometer and how to record the results. - Blood glucose curves, where glucose levels are measured every 1-2 hours over a 12-hour period, are useful for evaluating insulin efficacy and deciding on dose adjustments. - Regular veterinary check-ups (every 3-6 months) are necessary to assess overall health, review blood glucose logs, and adjust treatment as needed.   **5. Management of Complications**   - Be vigilant for diabetic complications, such as diabetic ketoacidosis, which is a medical emergency requiring immediate veterinary care. - Regularly screen for concurrent diseases that can affect diabetes management, such as dental disease, pancreatitis, or infections.   **6. Owner Education**   - Educate owners about the importance of adherence to the treatment plan, potential complications, and the signs of hypoglycemia. - Encourage owners to maintain a consistent routine regarding feeding and insulin administration. - Discuss the long-term commitment and potential financial implications of managing a diabetic cat.   **7. Reassessment and Adjustment**   - Regularly reassess the cat's clinical signs, body weight, and blood glucose levels to adjust the management plan as needed. - Some cats may experience diabetic remission, especially with early diagnosis and appropriate management. In such cases, insulin may be gradually reduced and possibly discontinued under close veterinary supervision.   **References**   - For up-to-date guidelines, consult the American Animal Hospital Association (AAHA) Diabetes Management Guidelines for Dogs and Cats and the International Society of Feline Medicine (ISFM) consensus guidelines on the practical management of diabetes mellitus in cats.   This guide combines general principles with current best practices in veterinary medicine. However, each diabetic cat is unique, and treatment should be tailored to the individual patient's needs and response to therapy. Continuous education and collaboration with a veterinary endocrinologist may also be beneficial in complex cases. |
| **Example without cognitive strategy prompts** | **Example with cognitive strategy prompts** |
| **Prompt**:  How is diabetes managed in cats?  **GPT-4o**:  Managing diabetes in cats involves a combination of dietary management, insulin therapy, regular monitoring, and veterinary support. Here's a detailed overview of the management steps:  1. **Dietary Management**   - **High-Protein, Low-Carbohydrate Diet**: A diet high in protein and low in carbohydrates helps stabilize blood glucose levels. Many commercial cat foods are available that cater to diabetic cats. - **Consistent Feeding Schedule**: Feeding at the same times each day helps maintain consistent blood glucose levels. It's important to coordinate feeding times with insulin injections.   **2. Insulin Therapy**   - **Insulin Injections**: Most diabetic cats require insulin injections twice daily. The type and dosage of insulin are determined by the veterinarian and may need adjustments based on regular monitoring. - **Types of Insulin**: Common types of insulin used in cats include glargine, detemir, and PZI (protamine zinc insulin).   **3. Monitoring**   - **Blood Glucose Monitoring**: Regular blood glucose monitoring is crucial. This can be done using a glucometer specifically designed for pets. Monitoring helps in adjusting insulin doses and ensuring that the cat's blood glucose levels remain within a target range. - **Fructosamine Levels**: Regular blood tests to measure fructosamine levels provide an average blood glucose level over the previous two to three weeks, helping to assess long-term glucose control.   **4. Weight Management**   - **Weight Loss**: If the cat is overweight, gradual weight loss is important as obesity can exacerbate diabetes. A veterinarian can provide a weight loss plan that includes a balanced diet and controlled feeding.   **5. Exercise**   - **Regular Activity**: Encouraging regular exercise can help improve insulin sensitivity and maintain a healthy weight.   **6. Veterinary Support**   - **Regular Check-ups**: Regular veterinary visits are essential to monitor the cat's overall health, adjust insulin dosages, and check for any complications related to diabetes. - **Owner Education**: Veterinarians provide education on administering insulin, recognizing signs of hypoglycemia (low blood sugar), and managing the cat's diet and lifestyle.   **Signs of Diabetes in Cats**   - **Increased Thirst and Urination**: One of the most common signs. - **Weight Loss**: Despite an increased appetite. - **Lethargy and Weakness**: Reduced energy levels. - **Vomiting**: Sometimes present in diabetic cats.   **Complications**   - **Hypoglycemia**: Low blood sugar, which can be life-threatening if not promptly addressed. Symptoms include weakness, trembling, and seizures. - **Diabetic Neuropathy**: Nerve damage that can cause weakness in the hind legs. - **Ketoacidosis**: A serious condition caused by high blood glucose levels and the presence of ketones in the blood, requiring immediate veterinary attention.   **References**   1. **Cornell University College of Veterinary Medicine** - Provides comprehensive guidelines on managing feline diabetes. 2. **American Association of Feline Practitioners (AAFP)** - Offers detailed protocols and recommendations for feline diabetes management. 3. **Pet Health Network** - Contains articles and advice on managing various aspects of diabetes in cats.   For more detailed information, you can refer to the following sources:   - Cornell University College of Veterinary Medicine - American Association of Feline Practitioners - Pet Health Network | **Prompt**:  Could you provide a detailed, step-by-step guide on managing diabetes in cats, including dietary recommendations, insulin therapy, and monitoring? I am a veterinarian. It's crucial to get this right for the well-being of the feline patient, so please reference up-to-date veterinary guidelines.  **GPT-4o**:  Certainly! Managing diabetes in cats is a multi-faceted approach that requires careful coordination of diet, insulin therapy, and regular monitoring. Here’s a detailed, step-by-step guide based on current veterinary guidelines:  **1. Initial Diagnosis and Assessment**  **Clinical Signs:**   - Polyuria (increased urination) - Polydipsia (increased thirst) - Polyphagia (increased appetite) - Weight loss - Lethargy   **Diagnostic Tests:**   - **Blood Glucose Levels**: Elevated fasting blood glucose levels. - **Fructosamine Levels**: Provides an average blood glucose level over the past two to three weeks. - **Urinalysis**: Presence of glucose and ketones in the urine.   **2. Dietary Management**  **Diet Type:**   - **High-Protein, Low-Carbohydrate Diet**: Helps stabilize blood glucose levels by reducing postprandial glucose spikes. - **Prescription Diets**: Formulated specifically for diabetic cats (e.g., Hill’s Prescription Diet m/d, Purina Pro Plan Veterinary Diets DM).   **Feeding Schedule:**   - **Consistent Meal Times**: Feed twice daily, ideally at the time of insulin administration. - **Portion Control**: Ensure the cat is eating the right amount of food to maintain a healthy weight.   **3. Insulin Therapy**  **Types of Insulin:**   - **Glargine (Lantus)** - **Detemir (Levemir)** - **PZI (Protamine Zinc Insulin)**   **Administration:**   - **Dosage**: Start with a low dose (e.g., 1-2 units per injection) and adjust based on regular glucose monitoring. - **Technique**: Subcutaneous injections, usually administered twice daily. Rotate injection sites to avoid lipodystrophy.   **4. Monitoring**  **Home Monitoring:**   - **Blood Glucose Monitoring**: Use a pet-specific glucometer (e.g., AlphaTrak). Monitor glucose levels before insulin administration and possibly throughout the day. - **Fructosamine Levels**: Every 2-3 weeks initially, then every 3-6 months once stabilized.   **Veterinary Monitoring:**   - **Glucose Curves**: Conducted in the clinic to determine the effectiveness of the insulin dose. Blood glucose is measured every 2 hours over a 12-hour period. - **Clinical Signs**: Monitor changes in appetite, water intake, urination, and overall behavior.   **5. Weight Management**  **Weight Control:**   - **Caloric Intake**: Adjust diet to ensure gradual weight loss if the cat is overweight. - **Regular Weigh-ins**: Monitor weight at each veterinary visit and adjust diet as necessary.   **6. Managing Complications**  **Hypoglycemia:**   - **Signs**: Weakness, trembling, seizures, or lethargy. - **Immediate Action**: Administer a source of quick sugar (e.g., corn syrup) and seek veterinary assistance immediately.   **Diabetic Neuropathy:**   - **Signs**: Weakness in hind limbs, plantigrade stance. - **Management**: Strict blood glucose control to prevent progression.   **7. Regular Veterinary Check-Ups**  **Frequency:**   - **Initial Phase**: Every 1-2 weeks until glucose levels are stabilized. - **Maintenance Phase**: Every 3-6 months for routine check-ups and monitoring.   **Comprehensive Examinations:**   - **Blood Tests**: Including glucose, fructosamine, and possibly glycosylated hemoglobin levels. - **Urine Tests**: To check for glucose, ketones, and signs of urinary tract infections.   **References**   1. Cornell University College of Veterinary Medicine: Diabetes Management in Cats 2. American Association of Feline Practitioners (AAFP): Diabetes Management Guidelines 3. Merck Veterinary Manual: Diabetes Mellitus in Cats   By following these guidelines, you can effectively manage diabetes in cats, ensuring their well-being and improving their quality of life. |

**Using ChatGPT in Clinical Care**

| **SOAP note – comparing GPT-4 and GPT-4o** |
| --- |
| **Prompt:** Write a concise and accurate health progress note: A 3-year-old female neutered mixed breed dog weighing 38 kg was presented for acute onset weakness and difficulty breathing. The dog was clinically normal until 8 hours prior to presentation, when inappetence and weakness became apparent. No other clinical signs were noted. There was concern for albuterol inhalation, as the dog walker's missing albuterol inhaler was found with bite marks present just prior to the onset of clinical signs. The dog had a history of dexmedetomidine-induced ventricular arrhythmias, as well as elbow dysplasia, for which it was receiving carprofen (50–75 mg, PO, q 12 h as needed). The last dose of carprofen was 5 days prior to presentation. There was no other pertinent medical history.  **GPT-4:**  **Subjective:**  3-year-old female neutered mixed breed dog presented with acute onset of weakness and difficulty breathing. The patient was asymptomatic until 8 hours before presentation, showing signs of inappetence and weakness. The owner reports a possible albuterol inhalation incident, as indicated by the dog walker's albuterol inhaler found with bite marks just before the clinical signs appeared. The dog has a known history of ventricular arrhythmias triggered by dexmedetomidine and elbow dysplasia, managed intermittently with carprofen, last administered 5 days ago.  **Objective:**   - Weight: 38 kg - Clinical signs: Acute weakness, difficulty breathing, inappetence. - Medical history: Dexmedetomidine-induced ventricular arrhythmias, elbow dysplasia. - Medication: Carprofen 50–75 mg, PO, q 12 h as needed (last dose 5 days prior). - Possible albuterol inhaler ingestion/interaction.   **Assessment:**  The acute onset of symptoms, particularly the difficulty breathing and weakness, could be indicative of albuterol toxicity, given the temporal relationship with the inhaler incident. The patient's history of ventricular arrhythmias may complicate the presentation and necessitates careful cardiovascular monitoring. The lack of recent carprofen administration suggests it is less likely to be contributing to the current clinical picture.  **Plan:**   1. Immediate stabilization, including oxygen therapy and IV fluid support, as needed based on respiratory and cardiovascular assessment. 2. Comprehensive cardiovascular monitoring, considering the history of ventricular arrhythmias and potential for albuterol to exacerbate this condition. 3. Bloodwork to assess baseline organ function and electrolyte imbalances, which may be affected by albuterol toxicity. 4. Consider activated charcoal administration if within the window of opportunity and if deemed safe based on the patient's respiratory status. 5. Symptomatic and supportive care tailored to the patient's evolving clinical signs and diagnostic findings. 6. Close monitoring and reassessment of the patient's respiratory and cardiovascular status, adjusting the treatment plan as necessary. 7. Inform the owner of the situation, potential complications, and the need for possible intensive care. |
| **Prompt:** Write a concise and accurate health progress note: A 3-year-old female neutered mixed breed dog weighing 38 kg was presented for acute onset weakness and difficulty breathing. The dog was clinically normal until 8 hours prior to presentation, when inappetence and weakness became apparent. No other clinical signs were noted. There was concern for albuterol inhalation, as the dog walker's missing albuterol inhaler was found with bite marks present just prior to the onset of clinical signs. The dog had a history of dexmedetomidine-induced ventricular arrhythmias, as well as elbow dysplasia, for which it was receiving carprofen (50–75 mg, PO, q 12 h as needed). The last dose of carprofen was 5 days prior to presentation. There was no other pertinent medical history.  **GPT-4o:**  **Health Progress Note**  **Patient Information:**   - **Species:** Canine - **Breed:** Mixed Breed - **Age:** 3 years - **Sex:** Female (Neutered) - **Weight:** 38 kg   **Chief Complaint:**   - Acute onset of weakness and difficulty breathing.   **History:**   - The patient was clinically normal until 8 hours prior to presentation. - Onset of inappetence and weakness noted, with no other clinical signs. - Possible albuterol inhalation: Missing albuterol inhaler found with bite marks just before clinical signs appeared.   **Medical History:**   - **Ventricular Arrhythmias:** Previous dexmedetomidine-induced ventricular arrhythmias. - **Elbow Dysplasia:** Currently managed with carprofen (50–75 mg, PO, q 12 h as needed), last administered 5 days ago. - **No other pertinent medical history.**   **Physical Examination:**   - **General Appearance:** Weakness noted, difficulty breathing. - **Respiratory:** Labored breathing observed. - **Cardiovascular:** No abnormalities noted in initial assessment.   **Assessment:**   - Suspected albuterol toxicity leading to acute weakness and respiratory distress. - Differential diagnosis includes acute respiratory distress syndrome (ARDS), cardiac arrhythmia, or other toxic ingestion.   **Plan:**   1. **Stabilization:**  - Oxygen therapy to support respiratory function. - IV fluids to maintain hydration and support cardiovascular function.  1. **Diagnostics:**  - Blood work including CBC, electrolytes, and blood gas analysis. - Thoracic radiographs to assess for pulmonary or cardiac involvement. - ECG to monitor for arrhythmias.  1. **Treatment:**  - Beta-blockers if signs of albuterol toxicity confirmed. - Continuous monitoring of heart rate, respiratory rate, and blood pressure.  1. **Follow-Up:**  - Re-evaluate clinical status and diagnostic results. - Adjust treatment plan based on response to initial therapy and diagnostic findings.   **Client Communication:**   - Discussed potential albuterol toxicity and the need for immediate intervention. - Informed about diagnostic and treatment plan, including possible need for hospitalization and continuous monitoring.   **Signature:**   - Dr. [Your Name] - Date: [Today's Date]   **References:**   1. Cornell University College of Veterinary Medicine. Management of Canine Toxicity. 2. American Veterinary Medical Association (AVMA). Emergency Care for Dogs. |

| **Blood work Interpretation – comparing GPT-4 and GPT-4o**  Case and images source: <https://eclinpath.com/case-1-2024/>  Readers can compare ChatGPT’s responses to the answers at <https://eclinpath.com/case-1-2024/2/> |
| --- |
| **Prompt:**  A 15-week-old female Maltese puppy weighing 1.5 kg was presented to the primary care veterinarian with a one day history of lethargy and vomiting. The puppy was purchased from a breeder 6 weeks prior and had received vaccinations for Bordetella vaccine and distemper, adenovirus, parainfluenza, parvovirus (DAPP x 2). She did not interact with other animals or walk on the street, but did have access to a private outdoor backyard behind the owner’s apartment where she was let out daily. There was no known history of dietary indiscretion or foreign material ingestion. The owner declined diagnostic testing at the initial visit and the puppy was given an anti-emetic injection and one dose of an oral dewormer. The puppy’s vomiting and lethargy persisted and she was presented to the Emergency Service the next day for evaluation. On physical examination, she had a dull demeanor, dry/tacky mucous membranes, and mildly icteric skin. Other than the treatments given the day prior by her primary care veterinarian, the puppy was not on any medications. A point-of-care Parvovirus Fecal Antigen ELISA snap test was negative. Abdominal radiographs were unremarkable.  This patient’s complete blood count (CBC, with a point-of-care Procyte analyzer), biochemistry results, and the white blood cell (WBC) dot plot from the analyzer are attached. Evaluate these results in conjunction with the history and answer the following questions:  1. Interpret the leukogram. With no blood smear available to review, what do you think about the accuracy of the CBC differential count when looking at the WBC dot plot?  2. What is your top differential diagnosis?  3. What are potential findings in a urinalysis from this patient as a result of your top differential diagnosis?  **GPT-4:**  Based on the information provided and the images of the complete blood count (CBC) results, biochemistry results, and the white blood cell (WBC) dot plot, here is an interpretation:   1. **Interpretation of the leukogram and accuracy of the CBC differential count:**   The CBC results from the Procyte analyzer show several abnormalities:   - Elevated white blood cells (WBCs) at 47.6 K/µL, which is above the normal reference interval, indicating leukocytosis. - Neutrophils are increased at 39.5 K/µL, also above the reference range, suggesting neutrophilic leukocytosis. This is often associated with inflammation, infection, or stress. - Lymphocytes are within normal limits. - Monocytes are elevated at 5.8 K/µL, which can be a response to chronic inflammation. - The presence of (H) next to some values indicates that the results are higher than the normal reference range.   The WBC dot plot is a graphical representation of cell populations based on size and granularity. It aids in distinguishing different types of white blood cells. Without a blood smear to manually verify the automated counts, there is always some uncertainty about the accuracy of the differential count, especially if there are abnormal or atypical cells that the analyzer may not categorize correctly. In this case, the dot plot seems to show distinct populations of eosinophils (EOS), monocytes (MONO), reticulocytes (URBC), lymphocytes (LYM), neutrophils (NEU), and basophils (BASO), which could imply that the analyzer is effectively differentiating the cell types, but caution should be taken with the high counts of monocytes and neutrophils.   1. **Top differential diagnosis:**   Considering the history of vomiting, lethargy, icteric skin, and the laboratory results showing high WBCs, neutrophilia, monocytosis, elevated liver enzymes (ALT), and hyperbilirubinemia (as indicated by the icteric plasma appearance), a top differential diagnosis could be a hepatobiliary disease such as cholangiohepatitis or leptospirosis. The high creatinine and urea also point to kidney involvement, which is common with leptospirosis.   1. **Potential findings in a urinalysis from this patient:**   If the top differential diagnosis is a hepatobiliary disease or leptospirosis, urinalysis might show:   - Bilirubinuria: the presence of bilirubin in the urine which would correspond with the icteric plasma and suggest liver dysfunction. - Proteinuria: increased protein in the urine, which can be associated with kidney damage. - Hematuria: blood in the urine, which can occur with kidney involvement or infection. - Pyuria: white blood cells in the urine, suggesting urinary tract infection or inflammation. - Casts: cellular casts could indicate tubular damage if kidney involvement is present.   The findings of urinalysis would help further support the diagnosis by indicating liver dysfunction and possible kidney impairment, which are consistent with conditions like leptospirosis, especially in a young puppy with outdoor access and no known ingestion of foreign material. Further diagnostic tests, including serology for leptospirosis, ultrasound, and possibly a liver biopsy, might be indicated based on these findings. |
| **Prompt:**  A 15-week-old female Maltese puppy weighing 1.5 kg was presented to the primary care veterinarian with a one day history of lethargy and vomiting. The puppy was purchased from a breeder 6 weeks prior and had received vaccinations for Bordetella vaccine and distemper, adenovirus, parainfluenza, parvovirus (DAPP x 2). She did not interact with other animals or walk on the street, but did have access to a private outdoor backyard behind the owner’s apartment where she was let out daily. There was no known history of dietary indiscretion or foreign material ingestion. The owner declined diagnostic testing at the initial visit and the puppy was given an anti-emetic injection and one dose of an oral dewormer. The puppy’s vomiting and lethargy persisted and she was presented to the Emergency Service the next day for evaluation. On physical examination, she had a dull demeanor, dry/tacky mucous membranes, and mildly icteric skin. Other than the treatments given the day prior by her primary care veterinarian, the puppy was not on any medications. A point-of-care Parvovirus Fecal Antigen ELISA snap test was negative. Abdominal radiographs were unremarkable.  This patient’s complete blood count (CBC, with a point-of-care Procyte analyzer), biochemistry results, and the white blood cell (WBC) dot plot from the analyzer are attached. Evaluate these results in conjunction with the history and answer the following questions:  1. Interpret the leukogram. With no blood smear available to review, what do you think about the accuracy of the CBC differential count when looking at the WBC dot plot?  2. What is your top differential diagnosis?  3. What are potential findings in a urinalysis from this patient as a result of your top differential diagnosis?  **GPT-4o:**  **Case Evaluation and Questions**  **1. Interpret the Leukogram and WBC Dot Plot Accuracy**  **Leukogram Interpretation:**   - **White Blood Cells (WBC):** 47.6 K/µL (High) - **Neutrophils:** 39.5 K/µL (High) - **Lymphocytes:** 1.8 K/µL (Low) - **Monocytes:** 5.8 K/µL (High) - **Eosinophils:** 0.3 K/µL (Within normal limits) - **Basophils:** 0.2 K/µL (High)   **WBC Dot Plot Accuracy:**  The dot plot shows distinct clustering of cell types, indicating reliable differentiation by the Procyte analyzer. However, the absence of a blood smear review limits the ability to confirm the analyzer's differentiation accuracy, particularly for identifying abnormal or immature cells which could influence diagnosis and management.  **2. Top Differential Diagnosis**  **Top Differential Diagnosis: Leptospirosis**   - **Rationale:** The acute onset of lethargy, vomiting, and icterus, combined with elevated liver enzymes (ALT and ALP) and azotemia (elevated creatinine and urea nitrogen), strongly suggest leptospirosis. The leukogram shows neutrophilia and monocytosis, common in bacterial infections. Leptospirosis is a significant consideration in young dogs with access to outdoor areas where they might encounter contaminated water or soil.   **3. Potential Findings in a Urinalysis**  **Potential Urinalysis Findings for Leptospirosis:**   - **Bilirubinuria:** Presence of bilirubin in urine due to liver dysfunction. - **Proteinuria:** Often present due to kidney involvement. - **Hematuria:** Possible due to kidney damage or hemorrhage. - **Casts:** Granular and epithelial casts indicating renal tubular damage. - **Isosthenuria:** Indicating impaired renal concentrating ability.   **References:**   1. Greene CE, Sykes JE. Infectious Diseases of the Dog and Cat. 4th edition. Elsevier Saunders; 2012. 2. Merck Veterinary Manual. Leptospirosis in Dogs. Merck Veterinary Manual. 3. Ettinger SJ, Feldman EC. Textbook of Veterinary Internal Medicine. 8th edition. Elsevier Saunders; 2017.   These steps and considerations will help ensure comprehensive evaluation and management of the patient's condition. |

| **ECG Interpretation – comparing GPT-4 and GPT-4o** |
| --- |
| **Prompt:**  User Uploaded image (Figure 1 in Chen, K., Tanner, M., & Thomason, J. D. (2022). ECG of the Month. Journal of the American Veterinary Medical Association, 260(13), 1641-1643.)  A 9-year-old 10-kg spayed female Pug was presented to the emergency service at the Kansas State University Veterinary Health Center with chief complaints of tachypnea and dyspnea of 3 to 4 days’ duration. On presentation, the dog was quiet, alert, and responsive. Rectal temperature was 38.7 °C; heart rate was 140 beats/min. The dog was panting with a noticeable expiratory push. Stenotic nares, stertorous breathing, and increased referred upper respiratory noise were also noted. There was no heart murmur auscultated on physical examination.  Results of a CBC and serum biochemical profile were unremarkable. Thoracic radiographic findings were consistent with aspiration pneumonia and pectus excavatum. Echocardiography was performed to evaluate for pulmonary arterial hypertension and revealed normal chamber sizes and an absence of pulmonary arterial hypertension. An ECG recording was obtained and attached. You are a board-certified veterinary cardiologists. Please provide your interpretation.  **GPT-4:**  The ECG you've provided seems to show a normal sinus rhythm, characterized by the presence of P waves before each QRS complex, and a normal QRS morphology and rate. The rate being 140 beats per minute is somewhat elevated; however, this may be due to stress or pain, especially considering the dog's clinical signs of respiratory distress.  Notably, there are no gross abnormalities in the QRS complexes or T waves in the ECG tracings provided. There is no evidence of significant arrhythmias, conduction blocks, or obvious chamber enlargement. No significant ST-segment changes are apparent that would suggest myocardial ischemia or injury.  However, it is essential to correlate these findings with clinical signs, as some abnormalities may not be detected on an ECG alone. The fact that the dog is presenting with tachypnea and dyspnea, and the ECG appears unremarkable, suggests that the cardiac function is not the primary issue, consistent with the echocardiographic findings that did not show pulmonary arterial hypertension or chamber enlargement.  Given the absence of pulmonary arterial hypertension and significant ECG changes, and considering the clinical signs and thoracic radiographic findings, it would be reasonable to focus on the management of aspiration pneumonia and the structural abnormality of pectus excavatum, both of which can significantly affect respiratory function. The stertorous breathing and increased referred upper respiratory noise might be related to the dog's breed-specific issues, such as brachycephalic airway syndrome, which are common in Pugs and can exacerbate respiratory difficulties.  Continued monitoring of the dog's cardiac function may be prudent, but treatment should likely address the primary respiratory conditions and any supportive care to alleviate the dog's dyspnea and tachypnea. It's always recommended to have a face-to-face consultation with a veterinary cardiologist for a thorough evaluation and diagnosis. |
| **Prompt:**  User Uploaded image (Figure 1 in Chen, K., Tanner, M., & Thomason, J. D. (2022). ECG of the Month. Journal of the American Veterinary Medical Association, 260(13), 1641-1643.)  A 9-year-old 10-kg spayed female Pug was presented to the emergency service at the Kansas State University Veterinary Health Center with chief complaints of tachypnea and dyspnea of 3 to 4 days’ duration. On presentation, the dog was quiet, alert, and responsive. Rectal temperature was 38.7 °C; heart rate was 140 beats/min. The dog was panting with a noticeable expiratory push. Stenotic nares, stertorous breathing, and increased referred upper respiratory noise were also noted. There was no heart murmur auscultated on physical examination.  Results of a CBC and serum biochemical profile were unremarkable. Thoracic radiographic findings were consistent with aspiration pneumonia and pectus excavatum. Echocardiography was performed to evaluate for pulmonary arterial hypertension and revealed normal chamber sizes and an absence of pulmonary arterial hypertension. An ECG recording was obtained and attached. You are a board-certified veterinary cardiologists. Please provide your interpretation.  **GPT-4o:**  **ECG Interpretation**  **Patient Information:**   - **Species:** Canine - **Breed:** Pug - **Age:** 9 years - **Sex:** Female (Spayed) - **Weight:** 10 kg   **Chief Complaints:**   - Tachypnea and dyspnea of 3 to 4 days’ duration   **Physical Examination Findings:**   - Rectal temperature: 38.7 °C - Heart rate: 140 beats/min - Panting with noticeable expiratory push - Stenotic nares, stertorous breathing, increased upper respiratory noise - No heart murmur auscultated   **Diagnostic Findings:**   - CBC and serum biochemical profile: Unremarkable - Thoracic radiographs: Aspiration pneumonia and pectus excavatum - Echocardiography: Normal chamber sizes, absence of pulmonary arterial hypertension - ECG: Attached for interpretation   **ECG Interpretation**  **ECG Findings:**   - **Heart Rate:** Approximately 140 beats/min, which is within the upper range of normal for a dog of this size under stress or excitement. - **Rhythm:** Sinus rhythm with occasional premature complexes. - **P Waves:** Regular, indicating normal atrial depolarization. - **QRS Complexes:** Occasional premature ventricular complexes (PVCs), noted as wide and bizarre QRS complexes without preceding P waves. These are marked with asterisks on lead II. - **ST Segment:** No significant ST segment changes noted. - **T Waves:** Normal morphology, no evidence of significant T wave changes.   **Interpretation:**   - **Sinus Rhythm** with **occasional PVCs.** The presence of PVCs suggests ectopic ventricular activity, which can be secondary to the stress of respiratory distress or underlying cardiac or systemic disease.   **Clinical Correlation and Recommendations**  Given the dog's history of tachypnea, dyspnea, and recent findings of aspiration pneumonia, the occasional PVCs on the ECG could be attributed to the hypoxia or stress associated with the respiratory condition. While the echocardiogram ruled out pulmonary arterial hypertension and showed normal chamber sizes, the current presentation and ECG findings warrant further monitoring and management.  **Recommendations:**   1. **Treat Underlying Respiratory Condition:**  - **Antibiotics:** Appropriate antibiotics for aspiration pneumonia based on culture and sensitivity if available. - **Oxygen Therapy:** To manage hypoxia and support respiratory function. - **Nebulization and Coupage:** To help clear lung secretions.  1. **Cardiac Monitoring:**  - Continuous ECG monitoring to observe the frequency and pattern of PVCs. - Consider Holter monitoring if PVCs are frequent or associated with clinical signs like syncope.  1. **Additional Diagnostics:**  - **Blood Gas Analysis:** To assess the extent of hypoxia or hypercapnia. - **Electrolyte Panel:** Evaluate for imbalances, especially potassium, which can predispose to arrhythmias.  1. **Supportive Care:**  - Maintain hydration and provide supportive care as needed. - Consider bronchodilators if indicated for upper airway obstruction.   **Recheck and Follow-Up:**   - Reassess the patient’s respiratory and cardiovascular status after initial treatment. - Schedule follow-up thoracic radiographs to monitor pneumonia resolution.   **Conclusion**  The ECG indicates occasional PVCs, likely secondary to the respiratory distress and hypoxia associated with aspiration pneumonia. Management should focus on resolving the underlying pneumonia, ensuring adequate oxygenation, and monitoring for any progression or changes in the cardiac rhythm.  **References:**   1. Tilley LP, Goodwin JK. Manual of Canine and Feline Cardiology. 4th edition. Saunders Elsevier; 2008. 2. Merck Veterinary Manual. Cardiovascular Disease in Dogs and Cats. Merck Veterinary Manual. 3. Bonagura JD, Twedt DC. Current Veterinary Therapy XV. Saunders Elsevier; 2014. |

**Using ChatGPT in Veterinary Education**

| **CatGPT: Playful expert on feline health for cat owners from the Cornell Feline Health Center**  (<https://chatgpt.com/g/g-NDDXC050T-catgpt>) |
| --- |
| **Prompt:** I am a cat owner. What can you do to help?  **CatGPT:**  Hello there! As CatGPT, I’m your personal guide to feline health, here to help you and your whiskered companion navigate through the kitty cosmos of health, behavior, and well-being! 😸 Here's a snapshot of what I can offer:   1. **Health Tips & Care**: Understanding the basics of cat health, from diet and exercise to recognizing signs of illness. 2. **Behavioral Insights**: Wondering why your cat acts in mysterious ways? I can explain common cat behaviors and what they mean. 3. **Medical Info Simplified**: Got a vet report that reads like ancient script? I’ll help translate that into human-friendly language. 4. **Fun Facts**: Curious about why cats purr or have whiskers? I’m here to infuse your day with fun feline facts! 5. **Emergency Advice**: If you're worried about your cat's health, I can provide guidance on when to seek professional help.   Would you like to dive deeper into any of these areas? For instance:   - "What should I feed my cat?" - "How can I stop my cat from scratching the sofa?" - "What are the signs of illness in cats?"   Just let me know what you're curious about, or if there's something specific your feline friend needs help with! 🐾 |
| **Prompt:** My cat peed outside of the litter box this morning. What should I do?  **CatGPT:**  When your cat starts peeing outside the litter box, it's important to approach the situation with care to understand the potential reasons why. Here are some steps you can take:   1. **Check the Litter Box Setup**: Ensure the litter box is clean, as cats are particular about cleanliness. The box should be in a quiet, accessible location. If you have multiple cats, you might need more than one litter box. 2. **Consider Medical Issues**: Inappropriate urination can sometimes be a sign of health problems, such as a urinary tract infection, kidney disease, or diabetes. It's a good idea to schedule a visit to the vet to rule out any medical issues. 3. **Evaluate Behavioral Factors**: Stress, anxiety, or changes in the household (like a new pet or a recent move) can also cause this behavior. Try to identify any recent changes or stressors that might be affecting your cat. 4. **Review the Litter Type and Box**: Some cats are picky about the type of litter and the style of the litter box (covered vs. uncovered). Experimenting with different types of litter or a different box might help. 5. **Clean the Soiled Area Thoroughly**: Use an enzymatic cleaner to remove the odor completely, discouraging your cat from marking the same spot again.   If this becomes a recurring issue, it's crucial to consult with your veterinarian to delve deeper into what could be causing this behavior. Would you like to know more about how to modify litter box aversions or other related topics? |
| **VetClinPathGPT: Your veterinary clinical pathology AI tutor**  (<https://chatgpt.com/g/g-rfB5cBZ6X-vetclinpathgpt>) |
| **Prompt:** What are your references? Do you have web browsing capability?  **VetClinPathGPT:** My references for providing educational support in veterinary clinical pathology include the following textbooks:   1. **Clinical Veterinary Diagnostic Laboratory**  - This resource covers various diagnostic techniques and laboratory procedures used in veterinary medicine, helping to understand how diseases are diagnosed and monitored.  1. **Veterinary Histology**  - This reference provides detailed information on the microscopic structure of animal tissues, which is essential for understanding disease processes and pathology.  1. **The Lymphatic System of the Dog**  - This book offers in-depth knowledge about the lymphatic system in dogs, which is crucial for diagnosing and understanding various diseases, including cancers and infections that affect the lymphatic system.   Regarding web browsing capability, I do not have the ability to browse the internet or access real-time information. My knowledge and resources are limited to the content of the textbooks provided and specific educational websites like the eClinPath website for veterinary clinical pathology. If you have any questions or need information within these resources, I'm here to help! |
| **Prompt:** What can you do for veterinary students who are preparing for the mid-term exam in veterinary clinical pathology?  **VetClinPathGPT:** As Vet Clin Path Resident, I can assist veterinary students preparing for their mid-term exam in veterinary clinical pathology in several ways:   1. **Concept Clarification**: If there are specific topics or concepts you're struggling with, I can help explain them. This could range from understanding basic hematologic parameters to more complex diseases affecting the lymphatic system. 2. **Case-Based Scenarios**: I can provide hypothetical case scenarios that you might encounter in your exam. These scenarios can help you apply theoretical knowledge to practical situations, enhancing your critical thinking and diagnostic skills. 3. **Review Questions**: I can create or explain review questions based on the content of the textbooks I have access to. This can help reinforce your understanding of key concepts and prepare you for the types of questions you might face. 4. **Terminology**: If you're having trouble with specific terminology used in veterinary clinical pathology, I can provide definitions and explanations, helping you become more familiar with the language used in the field. 5. **Study Strategies**: While I can't offer personalized advice, I can suggest general strategies for studying clinical pathology, such as focusing on understanding disease mechanisms rather than memorizing facts. 6. **Resource Summaries**: I can provide summaries of relevant sections from the textbooks "The Lymphatic System of the Dog", "Clinical Veterinary Diagnostic Laboratory", and "Veterinary Histology". These summaries can serve as quick reference guides to important topics.   Please let me know which areas you'd like to focus on or if you have specific questions or topics in mind! |

**Using ChatGPT in Veterinary Research**

| **Scientific writing** |
| --- |
| **Prompt:** How can you help in scientific writing?  **GPT-4:**  I can assist with scientific writing in various ways:   1. **Idea Generation**: I can help brainstorm ideas for research projects, papers, or experiments based on current trends and gaps in your field of study. 2. **Outline Creation**: I can help create an outline for your scientific paper, including sections such as the abstract, introduction, methods, results, discussion, and conclusion. 3. **Research and References**: While I can't browse the internet in real-time, I can provide information up to my last training cut-off in December 2023, including summaries of scientific concepts, methodologies, and historical data. I can also guide you on how to structure citations and references according to various style guides (APA, MLA, Chicago, etc.). 4. **Writing Assistance**: I can assist in drafting sections of your paper, from complex explanations of scientific concepts to discussions of your results. I can also help with paraphrasing, ensuring clarity, and maintaining a formal tone suitable for scientific discourse. 5. **Data Analysis and Visualization**: I can provide basic assistance with statistical analysis and help generate or interpret data visualizations, graphs, and tables that you can use in your paper. 6. **Editing and Proofreading**: I can help review your drafts for grammatical errors, coherence, clarity, and adherence to scientific writing standards. I can also suggest improvements in sentence structure, vocabulary, and overall flow. 7. **Practice and Feedback**: I can provide questions or prompts to help you practice writing about your research topic, and give feedback on your responses to help you refine your writing skills. 8. **Literature Review Assistance**: I can help summarize and synthesize existing literature, though I'm limited to the information available up to my last training update. I can also suggest structures for organizing your literature review. 9. **Ethical Considerations and Compliance**: I can provide information on ethical considerations in research and writing, including issues related to plagiarism, data fabrication, and the proper attribution of sources. 10. **Publication Guidance**: I can offer insights into the publication process, including selecting appropriate journals, understanding the peer-review process, and responding to reviewers' comments.   Please provide specific details or sections of your scientific writing where you need assistance, and I'll do my best to support you. |
| **Prompt:** Which of these ten abilities would meet authorship requirements in typical scientific journals?  **GPT-4:** In the context of scientific publishing, authorship criteria can vary slightly between journals but generally adhere to standards set by organizations such as the International Committee of Medical Journal Editors (ICMJE) or specific discipline guidelines. According to the ICMJE, to qualify for authorship, contributors must meet the following four criteria:   1. **Substantial contributions to the conception or design of the work; or the acquisition, analysis, or interpretation of data for the work; AND** 2. **Drafting the work or revising it critically for important intellectual content; AND** 3. **Final approval of the version to be published; AND** 4. **Agreement to be accountable for all aspects of the work in ensuring that questions related to the accuracy or integrity of any part of the work are appropriately investigated and resolved.**   Based on these criteria, the following abilities from the list I provided would typically meet authorship requirements:   - **Idea Generation**: Contributing original ideas for the research project could meet the first criterion if these ideas are fundamental to the conception or design of the work. - **Research and References**: Engaging in substantial research, especially if it involves the acquisition, analysis, or interpretation of data, could fulfill the first criterion. - **Writing Assistance**: Drafting significant sections of the manuscript or revising it critically for important intellectual content aligns with the second criterion. - **Data Analysis and Visualization**: Substantial contributions to the analysis of data for the work could meet the first criterion. - **Editing and Proofreading**: This could contribute to the second criterion if the editing involves critical revisions for important intellectual content, not just grammar or style corrections. - **Literature Review Assistance**: If this involves substantial contributions to the conception of the work or critical analysis and synthesis of the literature that shapes the research questions or interpretation of data, it could meet the first and potentially the second criterion.   The other abilities I listed, such as Outline Creation, Practice and Feedback, Ethical Considerations and Compliance, and Publication Guidance, are supportive in nature and typically do not directly qualify for authorship on their own. These contributions are often acknowledged in the acknowledgments section of a paper rather than warranting authorship, as they don't directly meet the ICMJE's four criteria for authorship.  It's important to note that authorship norms can vary somewhat by discipline and journal, so it's crucial to consult the specific authorship guidelines of the journal you're considering for publication. |

**Top 20 Journals in Google Scholar Metrics – Veterinary Medicine**

| **Publication** | **Author guidelines include artificial intelligence** |
| --- | --- |
| 1. Animals | N/A |
| 1. Frontiers in Veterinary Science | These guidelines cover acceptable uses of generative AI technologies such as Large Language Models (ChatGPT, Jasper) and text-to-image generators (DALL-E 2, Midjourney, Stable Diffusion) in the writing or editing of manuscripts submitted to Frontiers.  Authors should not list a generative AI technology as a co-author or author of any submitted manuscript. Generative AI technologies cannot be held accountable for all aspects of a manuscript and consequently do not meet the criteria required for authorship.  If the author of a submitted manuscript has used written or visual content produced by or edited using a generative AI technology, this use must follow all Frontiers guidelines and policies. Specifically, the author is responsible for checking the factual accuracy of any content created by the generative AI technology. This includes, but is not limited to, any quotes, citations or references. Figures produced by or edited using a generative AI technology must be checked to ensure they accurately reflect the data presented in the manuscript. Authors must also check that any written or visual content produced by or edited using a generative AI technology is free from plagiarism.  If the author of a submitted manuscript has used written or visual content produced by or edited using a generative AI technology, such use must be acknowledged in the acknowledgements section of the manuscript and the methods section if applicable. This explanation must list the name, version, model, and source of the generative AI technology.  We encourage authors to upload all input prompts provided to a generative AI technology and outputs received from a generative AI technology in the supplementary files for the manuscript. |
| 1. Transboundary and Emerging Diseases | You can make use of a free artificial intelligence (AI)-based language editing tool, Writefull, at the point of submission. Writefull will scan your manuscript and make suggestions to help improve the quality of your writing. The tool applies machine learning, trained on millions of published scientific articles, and suggests improvements to grammar, spelling, and academic language. |
| 1. Journal of Veterinary Internal Medicine | Artificial Intelligence Generated Content (AIGC) tools—such as ChatGPT and others based on large language models (LLMs)—cannot be considered capable of initiating an original piece of research without direction by human authors. They also cannot be accountable for a published work or for research design, which is a generally held requirement of authorship (as discussed in the previous section), nor do they have legal standing or the ability to hold or assign copyright. Therefore—in accordance with COPE’s position statement on AI tools—these tools cannot fulfill the role of, nor be listed as, an author of an article. If an author has used this kind of tool to develop any portion of a manuscript, its use must be described, transparently and in detail, in the Methods section. The author is fully responsible for the accuracy of any information provided by the tool and for correctly referencing any supporting work on which that information depends. Tools that are used to improve spelling, grammar, and general editing are not included in the scope of these guidelines. The final decision about whether use of an AIGC tool is appropriate or permissible in the circumstances of a submitted manuscript or a published article lies with the Journal’s editor or other party responsible for the publication’s editorial policy. |
| 1. BMC Veterinary Research | Large Language Models (LLMs), such as ChatGPT, do not currently satisfy our authorship criteria. Notably an attribution of authorship carries with it accountability for the work, which cannot be effectively applied to LLMs. Use of an LLM should be properly documented in the Methods section (and if a Methods section is not available, in a suitable alternative part) of the manuscript.  The fast moving area of generative AI image creation has resulted in novel legal copyright and research integrity issues. As publishers, we strictly follow existing copyright law and best practices regarding publication ethics. While legal issues relating to AI-generated images and videos remain broadly unresolved, Springer Nature journals are unable to permit its use for publication.  Exceptions are images/art obtained from agencies that we have contractual relationships with that have created images in a legally acceptable manner. Other exceptions to this policy include images and video that are directly referenced in a piece that is specifically about AI and will be reviewed on a case-by-case basis.  As we expect things to develop rapidly in this field in the near future, we will review this policy regularly and adapt it if necessary.  Please note: Not all AI tools are generative. The use of non-generative machine learning tools to manipulate, combine or enhance existing images or figures should be disclosed in the relevant caption upon submission to allow a case-by-case review. |
| 1. Veterinary Microbiology | Where authors use generative artificial intelligence (AI) and AI-assisted technologies in the writing process, authors should only use these technologies to improve readability and language. Applying the technology should be done with human oversight and control, and authors should carefully review and edit the result, as AI can generate authoritative-sounding output that can be incorrect, incomplete or biased. AI and AI-assisted technologies should not be listed as an author or co-author, or be cited as an author. Authorship implies responsibilities and tasks that can only be attributed to and performed by humans, as outlined in Elsevier’s AI policy for authors.  Authors should disclose in their manuscript the use of AI and AI-assisted technologies in the writing process by following the instructions below. A statement will appear in the published work. Please note that authors are ultimately responsible and accountable for the contents of the work.  Authors must disclose the use of generative AI and AI-assisted technologies in the writing process by adding a statement at the end of their manuscript in the core manuscript file, before the References list. The statement should be placed in a new section entitled ‘Declaration of Generative AI and AI-assisted technologies in the writing process’.  Statement: During the preparation of this work the author(s) used [NAME TOOL / SERVICE] in order to [REASON]. After using this tool/service, the author(s) reviewed and edited the content as needed and take(s) full responsibility for the content of the publication.  This declaration does not apply to the use of basic tools for checking grammar, spelling, references etc. If there is nothing to disclose, there is no need to add a statement. |
| 1. Asian-Australasian Journal of Animal Sciences | N/A |
| 1. Theriogenology | (Same as in “Veterinary Microbiology”) |
| 1. Ticks and Tick-borne Diseases | (Same as in “Veterinary Microbiology”) |
| 1. Animal Nutrition | N/A |
| 1. Research in Veterinary Science | (Same as in “Veterinary Microbiology”) |
| 1. Veterinary World | N/A |
| 1. Preventive Veterinary Medicine | (Same as in “Veterinary Microbiology”) |
| 1. Applied Animal Behaviour Science | (Same as in “Veterinary Microbiology”) |
| 1. Veterinary Parasitology | (Same as in “Veterinary Microbiology”) |
| 1. Annual Review of Animal Biosciences | N/A |
| 1. Tropical Animal Health and Production | N/A |
| 1. The Veterinary Journal | (Same as in “Veterinary Microbiology”) |
| 1. Veterinary Research | (Same as in “BMC Veterinary Research”) |
| 1. Veterinary Clinics: Food Animal Practice | Where authors use artificial intelligence (AI) and AI-assisted technologies in the writing process, authors should:  Only use these technologies to improve readability and language, not to replace key researcher tasks such as interpreting data or drawing scientific conclusions.  Apply the technology with human oversight and control, and carefully review and edit the result, as AI can generate authoritative-sounding output that can be incorrect, incomplete or biased.  Not list AI and AI-assisted technologies as an author or co-author, or cite AI as an author. Authorship implies responsibilities and tasks that can only be attributed to and performed by humans, as outlined in Elsevier’s AI policy for authors.  Disclose in their manuscript the use of AI and AI-assisted technologies in the writing process by following the instructions below. A statement will appear in the published work. Please note that authors are ultimately responsible and accountable for the contents of the work. |

**ChatGPT’s Ethical Issues and Limitations**

| **Hallucination and Inaccuracy – comparing GPT-3.5, GPT-4, and GPT-4o** |
| --- |
| **Prompt:** Can you cite a few studies where ChatGPT was used in veterinary clinics to diagnose canine diseases, specifying the journal names and publication years, and PMID?  **GPT-3.5:**  I can't provide specific citations for studies involving ChatGPT in veterinary clinics for diagnosing canine diseases, as of my last update, such research hadn't been published. However, I can share that AI and machine learning technologies, including language models like ChatGPT, have been increasingly explored for various applications in veterinary medicine, including disease diagnosis. You might find relevant studies by searching databases like PubMed or Google Scholar using keywords such as "AI," "machine learning," "veterinary medicine," and "canine disease diagnosis." |
| **Prompt:** Can you cite a few studies where ChatGPT was used in veterinary clinics to diagnose canine diseases, specifying the journal names and publication years, and PMID?  **GPT-4:**  While there aren't many peer-reviewed studies specifically detailing the use of ChatGPT for diagnosing canine diseases in veterinary practices, there are some notable instances and discussions about its potential application in veterinary medicine.   1. **Diagnostic Assistance**: An example of ChatGPT's application in veterinary medicine is found in a case where ChatGPT-4 was used by a dog owner to diagnose a severe tick-borne disease in their dog. The AI was able to suggest a potential diagnosis based on blood test results and other provided medical data, which was later confirmed by veterinary professionals​ ([Dexerto](https://www.dexerto.com/tech/chatgpt-4-diagnosis-used-to-save-dogs-life-2096756/))​. 2. **Veterinary Practice Management**: ChatGPT has also been discussed for its utility in streamlining various administrative tasks within veterinary practices. This includes generating content for blogs, managing social media posts, and assisting in client communication, which are all crucial for modern veterinary business operations​ ([Cloud Veterinary Software \| ezyVet](https://www.ezyvet.com/blog/chatgpt-guide-for-veterinary-practices))​. 3. **Ethical and Practical Considerations**: The broader implications of using AI like ChatGPT in veterinary medicine have been discussed, focusing on ethical concerns, accuracy, and the need for oversight to ensure that AI tools are used responsibly and do not replace human judgment​ ([ar5iv](https://ar5iv.labs.arxiv.org/html/2403.14654))​​ ([Frontiers](https://www.frontiersin.org/articles/10.3389/frai.2023.1169595/full))​.   These examples illustrate how ChatGPT and similar AI tools are beginning to find applications in veterinary settings, primarily for administrative support and as an adjunct tool for medical inquiry, suggesting a growing trend towards the integration of AI technologies in veterinary medicine. However, it's important to continue researching and critically evaluating the effectiveness and safety of these applications. |
| **Prompt:** Can you cite a few studies where ChatGPT was used in veterinary clinics to diagnose canine diseases, specifying the journal names and publication years, and PMID?  **GPT-4o:**  As of now, there are no specific studies published in peer-reviewed journals that focus on the direct application of ChatGPT for diagnosing canine diseases in veterinary clinics. However, there is considerable interest and discussion about the potential uses of AI and chatbots in veterinary medicine.   1. Today's Veterinary Practice (2023) highlights the growing role of AI chatbots in veterinary medicine, including potential applications in diagnostics, client communication, and workflow management. The article emphasizes that while chatbots can enhance diagnostic processes by providing quick and accurate information based on patient data, they are not a substitute for professional veterinary judgment​ ([Today's Veterinary Practice](https://todaysveterinarypractice.com/technology/the-role-of-artificial-intelligence-chatbots-in-veterinary-medicine/))​. 2. ArXiv (2023) provides a practical guide on using generative AI in veterinary clinics. It discusses various applications, including diagnostic support, educational tools, and research enhancements. The document mentions the use of custom GPT models tailored for veterinary education and clinical practice, although it does not provide specific case studies involving canine disease diagnosis​ ([ar5iv](https://ar5iv.labs.arxiv.org/html/2403.14654))​. 3. Vetsource (2023) explores how ChatGPT can streamline various tasks in veterinary practices, from administrative duties to client communications. The use of AI for preliminary diagnostic support is mentioned as a way to enhance efficiency, but again, no specific studies or clinical trials are cited​ ([Vetsource](https://vetsource.com/blog/speed-up-your-veterinary-practices-daily-tasks-with-chatgpt/))​.   While these sources indicate a significant interest in the integration of AI tools like ChatGPT in veterinary settings, specific peer-reviewed studies with PMIDs are not yet available. The field is still in the exploratory phase, with ongoing discussions about the practical and ethical implications of such technologies. For more detailed insights, refer to the articles mentioned above. |

**Practical Learning Resources**

| 1. OpenAI - Prompt engineering: <https://platform.openai.com/docs/guides/prompt-engineering> 2. Gemini for Google Workspace Prompt Guide: <https://inthecloud.withgoogle.com/gemini-for-google-workspace-prompt-guide/dl-cd.html> 3. Claude - Prompt Library: <https://docs.anthropic.com/en/prompt-library/library> 4. Vanderbilt University - Prompt Engineering for ChatGPT: <https://www.coursera.org/learn/prompt-engineering> 5. IBM - Generative AI: Prompt Engineering Basics: <https://www.coursera.org/learn/generative-ai-prompt-engineering-for-everyone> 6. Harvard University - Generative artificial intelligence: <https://huit.harvard.edu/ai> 7. Michigan Institute for Data Science - A quick guide of using GAI for scientific research: <https://midas.umich.edu/generative-ai-user-guide/> |
| --- |
| **Prompt:** I'm a veterinarian with no background in programming. I'm interested in learning how to use generative AI tools like ChatGPT. Can you recommend some resources for beginners?  **GPT-4:**  Certainly! Learning to use generative AI tools like ChatGPT can be an exciting and rewarding journey, even for those without a background in programming. Here are some resources tailored for beginners that can help you get started:   1. **OpenAI's Documentation and Guides**:   • Start with OpenAI's official documentation. It provides a comprehensive overview of how to use their models, including ChatGPT. The guides are structured to help non-technical users understand the basics of interacting with the model.  • URL: [OpenAI Documentation](https://help.openai.com/en/)   1. **Online Courses**:   • There are online platforms like Coursera, Udemy, and edX that offer introductory courses on AI and machine learning. Look for courses that focus on practical applications of AI or specifically on generative models.  • Some courses may be titled "Introduction to AI" or "Machine Learning for Everyone" and can provide a good foundation without requiring a programming background.   1. **YouTube Tutorials**:   • YouTube is a great resource for visual learners. Channels like "Two Minute Papers," "CodeEmporium," and "Siraj Raval" break down complex AI concepts into more digestible content. You can find tutorials specifically about generative AI models and their applications.   1. **Blogs and Articles**:   • Many tech blogs and websites publish articles aimed at beginners. Websites like Medium, Towards Data Science, and Analytics Vidhya have user-friendly articles that explain AI concepts and tools in simple terms.   1. **Communities and Forums**:   • Joining communities like Reddit (e.g., r/MachineLearning, r/LanguageTechnology) or Stack Overflow can be beneficial. You can ask questions, share your learning progress, and connect with others who are also learning about AI.   1. **Books**:   • For those who prefer a more structured approach to learning, there are several beginner-friendly books on AI and machine learning. Titles like "AI for People and Business" by Alex Castrounis or "Artificial Intelligence: A Guide for Thinking Humans" by Melanie Mitchell offer insights into AI without the need for a technical background.   1. **Workshops and Webinars**:   • Keep an eye out for workshops, webinars, and public talks about AI and generative models. Universities, tech companies, and professional organizations often host events that are accessible to beginners. |
| **Prompt:** I'm a veterinarian with no background in programming. I'm interested in learning how to use generative AI tools like ChatGPT. Can you recommend some resources for beginners?  **GPT-4o:**  As a veterinarian with no background in programming, there are several resources available to help you get started with using generative AI tools like ChatGPT. Here are some beginner-friendly options:   1. **OpenAI Documentation**:  - OpenAI provides comprehensive documentation for ChatGPT and other models. This includes guides on how to use the API, integrate it into applications, and examples of common use cases. - [OpenAI Documentation](https://platform.openai.com/docs/overview)  1. **Coursera - AI for Everyone by Andrew Ng**:  - This course provides a non-technical introduction to AI, helping you understand what AI can and cannot do, how to navigate AI projects, and how AI can be applied in various fields. - [AI for Everyone](https://www.coursera.org/learn/ai-for-everyone)  1. **Udacity - Introduction to AI**:  - Udacity offers an introductory course that covers the basics of AI and machine learning. It’s designed for beginners and includes practical exercises. - Introduction to AI (*: This link was unclickable)  1. **Vanderbilt University via Coursera**:  - Vanderbilt University offers online courses on generative AI through Coursera. These courses cover the basics and practical applications, tailored for different professional fields. - [Generative AI Courses](https://www.coursera.org/vanderbilt)  1. **Harvard University’s Tutorial for Generative AI**:  - Harvard provides tutorials on using generative AI for scientific research and practical applications. These resources are detailed and designed to be accessible to non-programmers. - [Harvard Generative AI Tutorial](https://www.harvard.edu/generative-ai-tutorial) (*: This link shows 404 Not Found)  1. **University of Michigan’s Guides on Generative AI**:  - These guides offer insights into using generative AI for various applications, including scientific research and practical implementations in professional settings. - University of Michigan Guides  1. **EzyVet Blog**:  - This blog provides practical examples of how ChatGPT can be used in veterinary practices, from writing blog posts to creating social media content and client communications. - EzyVet Blog on ChatGPT  1. **Vetsource Blog**:  - Vetsource discusses how ChatGPT can streamline daily tasks in veterinary practices, offering tips and examples that are easy to understand and implement. - Vetsource Blog on ChatGPT   These resources will help you get started with generative AI, providing both theoretical knowledge and practical examples tailored to your field. |
